# APOBEC3B and CD274 as Combined Biomarkers for Predicting Response to Immunotherapy in Urothelial Carcinoma of the Bladder

**DOI:** 10.1155/2022/6042334

**Published:** 2022-09-24

**Authors:** Chuanhao Zhang, Zhichao Cheng, Zhe Wang, Genghao Zhao, Yonghui Yuan, Ruoyu Wang

**Affiliations:** ^1^Graduate School of Dalian Medical University, Dalian, China; ^2^Departement of Medical Oncology, Affiliated Zhongshan Hospital of Dalian University, Dalian, China; ^3^The Key Laboratory of Biomarker High Throughput Screening and Target Translation of Breast and Gastrointestinal Tumor, Dalian University, Dalian, China; ^4^Graduate School of Xuzhou Medical University, Xuzhou, China; ^5^Cancer Hospital of China Medical University Liaoning Cancer Hospital & Institute, Shenyang, China

## Abstract

Immunotherapy has become a promising form of treatment for cancers. There is a need to predict response to immunotherapy accurately. In the UCSC Xena, pan-cancer analysis revealed a positive relationship between APOBEC3B (A3B) and tumor mutational burden (*R* = 0.28, *P* < 0.001) and microsatellite instability (*R* = 0.12, *P* < 0.05). Naturally, the A3B high expression group had higher tumor mutational burden and microsatellite instability than the low expression group. The bladder cancer (BLCA) cohort in The Cancer Genome Atlas (TCGA) revealed tumor mutational signatures of A3B high and low expression groups. Compared to the low expression group, the high expression group had a higher number of SNPs and mutations. Subsequently, A3B was profiled for immune cell infiltration and immune checkpoints in bladder cancer. The results showed that A3B was positively correlated with most immune cells. Compared with the A3B low expression group, the A3B high expression group had higher expression of immune checkpoints. A3B was positively correlated with CD274 (*R* = 0.12, *P* = 0.016). This indicated that the high expression of A3B may have a better response to immunotherapy. Furthermore, data from the IMvigor210 immunotherapy clinical trial was used to confirm the findings of this study. The combined survival analysis of A3B and CD274 showed that the group of patients with high expression of CD274 and A3B was found to have a significantly higher survival rate than the rest of the patient group (*P* < 0.047). The results demonstrated that A3B has a significant role in immunotherapy. Moreover, the combined biomarkers of A3B and CD274 were more effective in predicting response to immunotherapy in bladder urothelial carcinoma. The findings of this study provide valuable insights for precision medicine.

## 1. Introduction

Bladder cancer is the 11th most common cause of cancer-related deaths worldwide [1]. In recent years, immunotherapy has emerged as a promising cancer treatment modality. It is essential to have a thorough understanding of the heterogeneity of the tumor microenviroment [2]. Tumor mutational burden (TMB), microsatellite instability (MSI), and programmed death-ligand 1 (PD-L1) could be used as predictive markers to identify patients likely to benefit from immunotherapy [3–6]. Immunotherapy targeting single biomarkers have been demonstrated to be less effective than that targeting composite biomarkers. Therefore, there is a need to explore combined biomarkers that could be targeted to improve the effectiveness of immunotherapy.

Human apolipoprotein B mRNA editing catalytic polypeptide-like 3B (APOBEC3B), one of the seven members of the cytidine deaminase family, is upregulated in multiple cancer types [7, 8]. Furthermore, the APOBEC3B (A3B) mutational signature is enriched in various cancer types, including the bladder, breast, cervical, and thyroid cancers [9, 10]. A high tumor mutation burden is used as a predictive biomarker to identify patients who may benefit from immune checkpoint inhibitors (ICIs). According to a previous study, A3B was positively correlated with PD-L1 expression and T-cell infiltration [11]. Therefore, we hypothesized that A3B could be a potential biomarker for predicting response to immunotherapy.

Using bioinformatics tools, we explored the correlation between A3B and response to immunotherapy. Further, we explored the significance of combined biomarkers of A3B and PD-L1 (CD274) against CD274 alone in predicting response to immunotherapy.

## 2. Materials and Methods

### 2.1. Data Collection

Pan-cancer RNA sequencing and clinical data from 33 types of cancers were downloaded from the UCSC Xena database (http://xena. ucsc.edu/). In addition, mutation data were obtained from the GDC website (https://portal.gdc.cancer.gov/). Data from the IMvigor210 clinical trial were downloaded through the “IMvigor210CoreBiology” R package (http://research-pub.gene.com/IMvigor210CoreBiologies/).

### 2.2. Differential Expression of A3B

Differential expression of APOBEC3B genes between tumor and paracancerous tissues was conducted using the “ggpubr” R package. The results were presented in boxplots. The tumors were staged as stage 0, stage I, stage II, stage III, and stage IV. Stages 0, I, and II were defined as early stages, while stages III and IV were defined as late stages. Boxplots were used to show differences between the early and late stages of tumors.

### 2.3. Tumor Mutation Signature of A3B

The association between A3B and MSI or TMB in pan-cancer was drawn using the “fmsb” R package. The results were presented in radar charts. Data in the TCGA-BLCA dataset were divided into high and low expression groups based on the median A3B expression value. The expression of MSI and TMB between the A3B high- and low-expression groups were compared using the “limma” R package [12]. The “maftools” R package [13] was used to visualize and draw waterfall plots showing mutational signatures between the high and low expression groups of A3B.

### 2.4. Immune Correlation Analysis

Immunophenotyping data were downloaded from the UCSC Xena website (Supplementary Table 1). C1 means wound healing, C2 means IFN-gamma dominant, C3 means inflammatory, C4 means lymphocyte depleted, C5 means immunologically quiet, and C6 means TGF-beta dominant. After that, boxplots showing the different immunophenotypes were drawn. Data on immune cell infiltration were obtained from the TIMER2.0 online website (http://timer.comp-genomics.org/) [14–16]. The correlation between A3B and immune cells was visualized using the “tidyverse” R package. Further, differences in immune checkpoints between the high and low expression groups were compared by the Wilcoxon test.

### 2.5. Immunotherapy Response

Data from the IMvigor210 immunotherapy cohort, including 195 patients with bladder urothelial carcinoma with complete information on survival, were analyzed. Spearman's correlation was used to show the correlation between A3B and CD274. Further, patients were stratified into four groups based on the expression of A3B and CD274, including A3B high CD274 high expression group, A3B high CD274 low expression group, A3B low CD274 low expression group, and A3B low CD274 high expression group. Survival analysis was done using the “survival” R package.

### 2.6. Statistical Analysis

All statistical analyses were performed using R statistical software (version 4.0.3). The Kruskal-Wallis test was used to analyze differences in the distribution of the immune cell types. Spearman's correlation was used for the correlation between A3B and CD274. Kaplan-Meier survival analysis was used to analyze survival between groups. The *P* values <0.05 (^∗^), 0.01 (^∗∗^), and 0.001 (^∗∗∗^) were considered statistically significant.

## 3. Results

### 3.1. Differential Expression of A3B in Pan-Cancer

**Figure 1:**
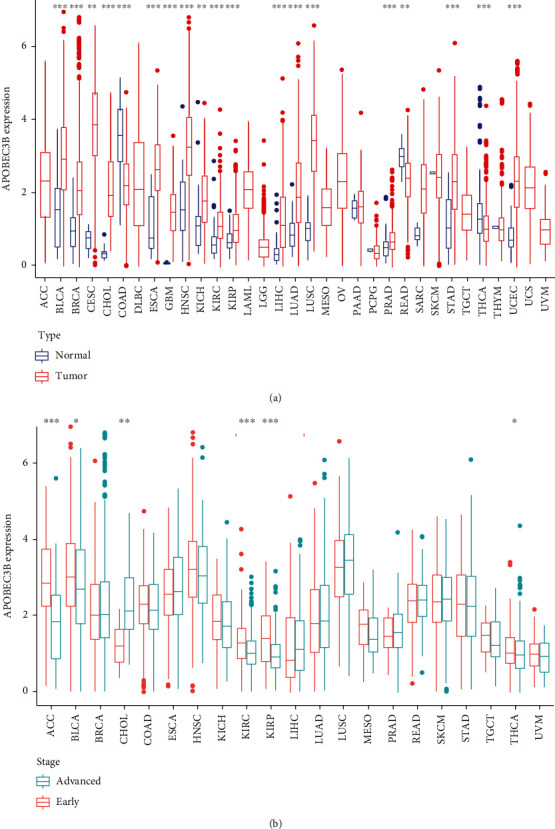
Differential expression of A3B in pan-cancer. (a) Boxplots showing the differential expression of A3B in normal and tumor tissues in pan-cancer. (b) Boxplots showing the differential expression of A3B in the early and late stages of pan-cancer. ^∗^*P* < 0.05, ^∗∗^*P* < 0.01, ^∗∗∗^*P* < 0.001.

The differential expression analysis of A3B among the 33 types of cancers showed significant differences in the expression of A3B in bladder cancer (BLCA), breast cancer (BRCA), cervical squamous cell carcinoma and endocervical adenocarcinoma (CESC), colon adenocarcinoma (COAD), esophageal cancer (ESCA), glioblastoma multiforme (GBM), head and neck squamous cell carcinoma (HNSC), kidney chromophobe (KICH), kidney renal clear cell carcinoma (KIRC), kidney renal papillary cell carcinoma (KIRP), liver hepatocellular carcinoma (LIHC), lung adenocarcinoma (LUAD), lung squamous cell carcinoma (LUSC), prostate cancer (PRAD), rectal adenocarcinoma (READ), stomach adenocarcinoma (STAD), thyroid carcinoma (THCA), and uterine corpus endometrial carcinoma (UCEC) (Figure 1(a)). Similarly, the differential expression analysis of A3B in the different pathological stages (early stages: stages 0, I, and II; and advanced stages: stages III and IV) revealed significant differences in adrenal cortex carcinoma (ACC), bladder cancer (BLCA), cholangiocarcinoma (CHOL), kidney renal clear cell carcinoma (KIRC), kidney renal papillary cell carcinoma (KIRP), and thyroid carcinoma (THCA) (Figure 1(b)).

### 3.2. MSI and Tumor Mutational Signatures of A3B

**Figure 2:**
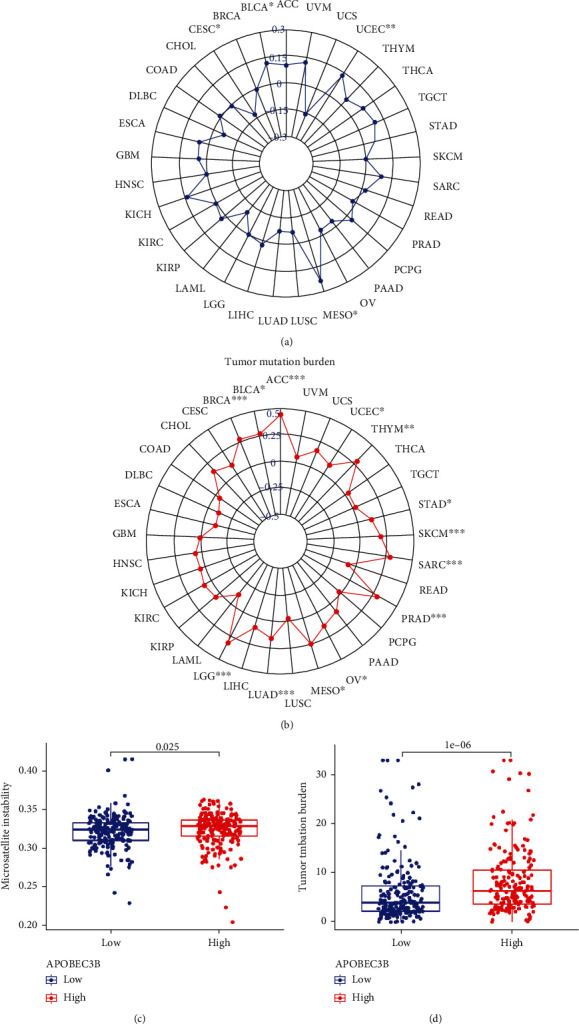
Correlation between A3B, TMB, and MSI. (a) Correlation between A3B and MSI in pan-cancer. (b) Differences in the expression of MSI between the high and low A3B expression groups in the TCGA-BLCA dataset. (c) Correlation between A3B and TMB in pan-cancer. (d) Differences in the expression of TMB between the high and low A3B expression groups in the TCGA-BLCA dataset. ^∗^*P* < 0.05, ^∗∗^*P* < 0.01, ^∗∗∗^*P* < 0.001.

**Figure 3:**
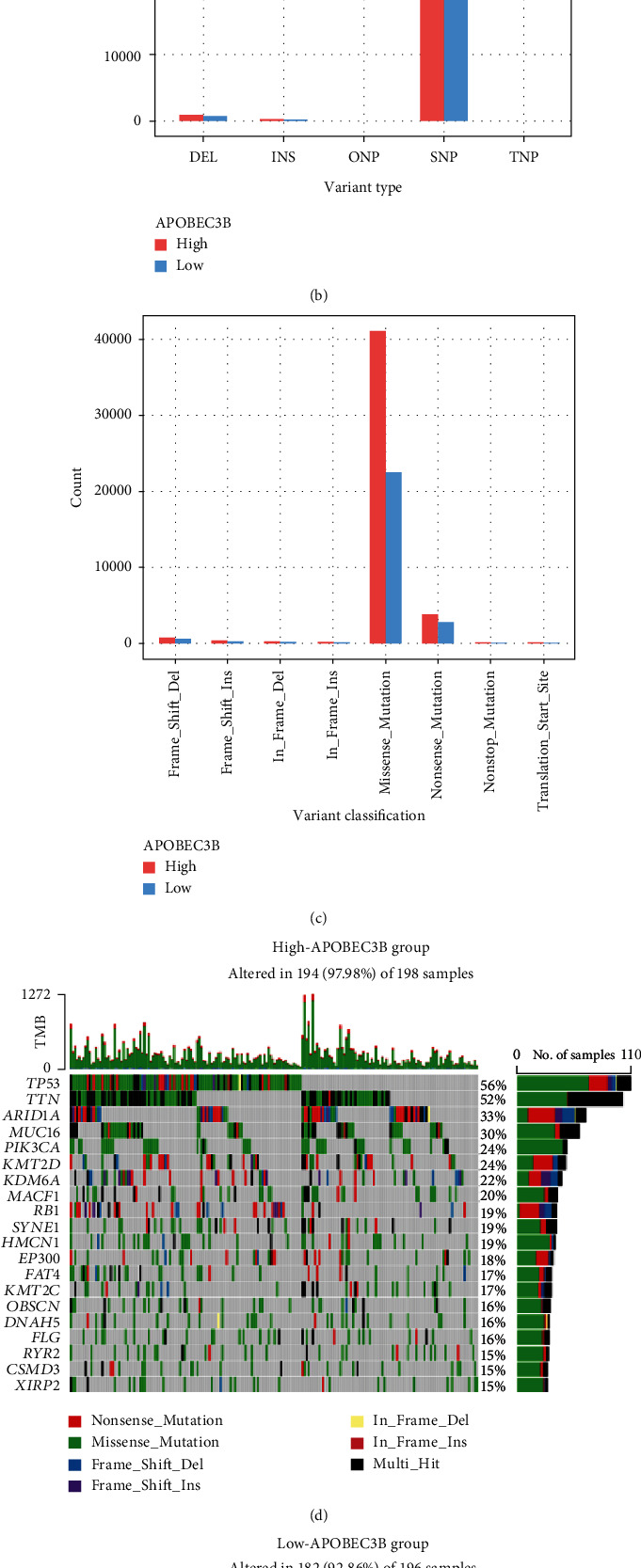
Mutations in the high and low A3B expression groups in the TCGA-BLCA. (a) Mutations in the A3B high expression group. (b) Mutations in the A3B low expression group. (c) Waterfall plot showing the top 20 mutated genes in the A3B high expression group. (d) Waterfall plot showing the top 20 mutated genes in the A3B low expression group.

TMB and MSI are predictive markers for immunotherapy response. The correlation between MSI and pan-cancer was shown on a radar chart. A3B and MSI were shown to be correlated in several cancer types, including BLCA (*R* = 0.12, *P* < 0.05), CESC (*R* = −0.13, P < 0.05), MESO (*R* = 0.24, P < 0.05), and UCEC (*R* = 0.13, P < 0.01) (Figure 2(a)). In addition, as shown in Figure 2(b), A3B was significantly correlated with TMB in ACC (*R* = 0.44, *P* < 0.001), BLCA (*R* = 0.28, *P* < 0.001), BRCA (*R* = 0.28, *P* < 0.001), LGG (*R* = 0.32, *P* < 0.001), LUAD (*R* = 0.16, *P* < 0.001), MESO (*R* = 0.25, *P* < 0.05), OV (*R* = 0.14, P < 0.05), PRAD (*R* = 0.29, *P* < 0.001), SARC (*R* = 0.29, *P* < 0.001), SKCM (*R* = 0.19, *P* < 0.001), STAD (*R* = 0.13, *P* < 0.05), THYM (*R* = 0.29, *P* < 0.01), and UCEC (*R* = 0.10, *P* < 0.05). A3B was associated with TMB and MSI in BLCA, MESO, and UCEC. The only in-depth analysis we performed was on bladder cancer to facilitate the study of immunotherapy. Therefore, we analyzed the differential expression of MSI between the high and low A3B expression groups in the TCGA-BLCA dataset. As shown in Figure 2(a), A3B was positively correlated with MSI. Furthermore, the A3B high expression group had a higher expression of MSI (Figure 2(c)). Similarly, the A3B high expression group had a higher expression of TMB (Figure 2(d)). Data were divided into A3B high and low expression groups based on the median cutoff value. The single nucleotide polymorphism and C > T were the most prevalent type in the TCGA-BLCA dataset (Figures 3(a) and 3(b)). Furthermore, missense mutations were shown to be the primary mutations in the A3B high expression group (Figure 3(c)). However, the above indicators of the A3B low expression group were lower than those of the A3B high expression group (Figures 3(a)–3(c)). The waterfall plot revealed that mutations are more prevalent in the high-expression A3B group compared to the low-expression A3B group (Figures 3(d) and 3(e)).

### 3.3. Immune Correlation Analysis of Pan-Cancer and Bladder Cancer

**Figure 4:**
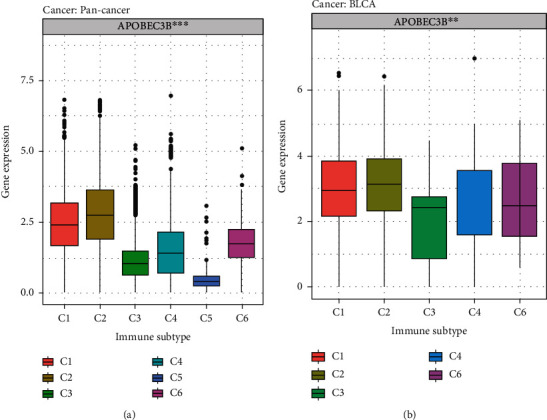
Immunophenotyping in pan-cancer and the TCGA-BLCA. (a) Immunophenotyping of A3B in pan-cancer. (b) Immunophenotyping of A3B in bladder cancer. C1: wound healing; C2: IFN-gamma dominant; C3: inflammatory; C4: lymphocyte depleted; C5: immunologically quiet; C6: TGF-beta dominant. ^∗∗^*P* < 0.01, ^∗∗∗^*P* < 0.001.

In recent years, immunotherapy has become a promising treatment modality for cancer patients. The expression of A3B in each immunophenotype was analyzed based on pan-cancer immunophenotypic data (Figure 4(a)). Notably, A3B was significantly higher in C1 (wound healing) and C2 (IFN-gamma dominant) than the other immunotypes. However, the expression of A3B was significantly low in C5 (immunologically quiet). Similarly, analysis of A3B expression among the different immune cell types in the TCGA-BLCA dataset revealed a significantly high expression of A3B in C1 and C2 cell types and a significantly low expression of A3B in C5 (Figure 4(b)). These results revealed an association between A3B and cellular immunity. There was no data available on C5 typing in the TCGA-BLCA dataset.

**Figure 5:**
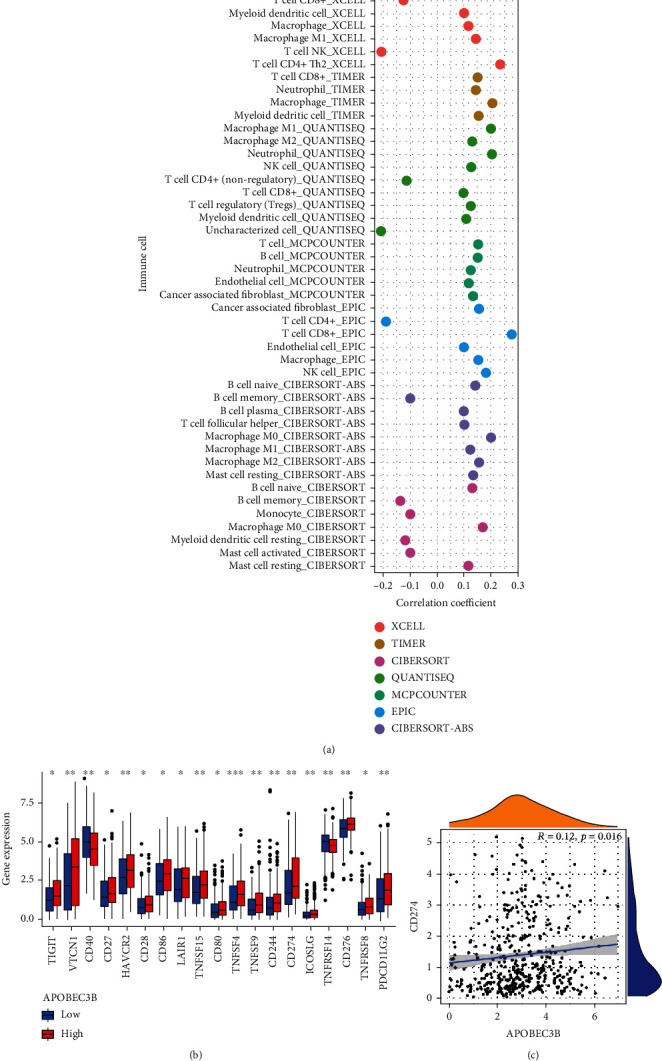
Immune correlation analysis of A3B in the TCGA-BLCA cohort. (a) Scatter plot showing the correlation between A3B and immune cells. (b) Boxplots showing differences in immune checkpoints between the high and low A3B expression groups. (c) Correlation between A3B and CD274. ^∗^*P* < 0.05, ^∗∗^*P* < 0.01, ^∗∗∗^*P* < 0.001.

Further, we explored the relationship between A3B and immune cell infiltration, immune checkpoints, and CD274. The relationship between immune cells and A3B was shown on a bubble chart. The majority of immune cells were positively correlated with A3B, including CD8+ T cells, B cells, macrophages, and NK cells, while a few were negatively correlated. Overall, the A3B high expression group usually had a large amount of immune cell infiltration. Immunotherapy is undoubtedly more effective in an environment with high immune cell infiltration (Figure 5(a)). In addition, several immune checkpoints, including CD274, HAVCR2, and TIGIT were highly expressed in the A3B high expression group (Figure 5(b); Supplementary Table 2). Finally, there was a significant correlation between A3B and CD274 (Figure 5(c)) (*R* = 0.12, *P* = 0.016). These results reveal that A3B plays an essential role in immunotherapy. Since A3B is closely related to immunotherapy response markers such as MSI, TMB, and CD274, we hypothesized that A3B could be exploited as marker for immunotherapy response.

### 3.4. Validation of A3B in an Immunotherapy Cohort

**Figure 6:**
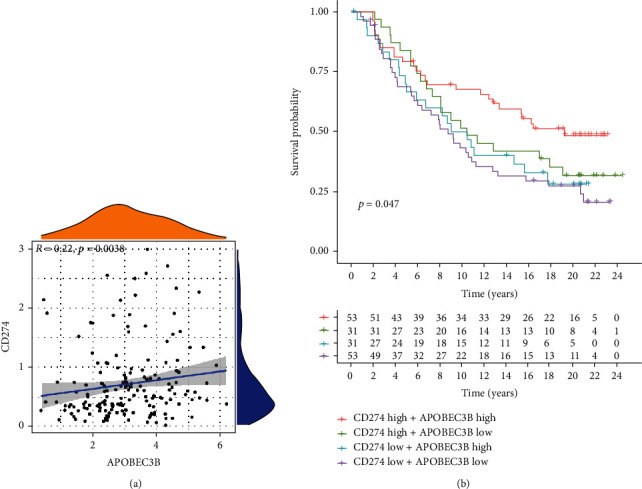
Validation of A3B in the IMvigor210 immunotherapy cohort. (a) Spearman correlation of A3B and CD274. (b) Survival analysis of the A3B combined with CD274 group. A3B and CD274 were divided into high and low expression groups based on the median value.

Next, we analyzed the publicly available data from the IMvigor210 immunotherapy cohort, which included 195 patients with bladder urothelial carcinoma. The results revealed that A3B was associated with CD274 in the immunotherapy cohort (*R* = 0.22, *P* = 0.038) (Figure 6(a)). In addition, the survival analysis revealed that the patient group with a high expression of CD274 and A3B had significantly higher survival rate than the patient group with low expression of CD274 and A3B (Figure 6(b)). In addition, the patient group with a high expression of CD274 and A3B had better survival rate than the patient group with a high expression of CD274 and a low expression of A3B. These results suggested that immunotherapy targeting both CD274 and A3B could be more effective than immunotherapy targeted against a single biomarker. Moreover, the overall survival analysis revealed that the CD274 and A3B low expression group had significantly lower survival than the CD274 low expression and A3B high expression group. Furthermore, the overall survival of the CD274 high expression and A3B high expression group was significantly higher than that of the CD274 low expression group and A3B high expression group. Taken together, these results revealed that immunotherapeutic agents targeting A3B and CD274 would be more effective than those targeting only one biomarker.

## 4. Discussion

Accumulating evidence has shown that A3B plays significant role in immunity, including tumor mutational signatures, immune cell infiltration, immune checkpoints, and immunotherapy. Therefore, to determine the role of A3B as a predictive biomarker for immunotherapy response in individual cancers, we conducted a preliminary exploration of A3B in pan-cancer. Notably, A3B was shown to be a predictive biomarker for immunotherapy response in bladder cancer. Furthermore, immunotherapy targeting composite biomarkers is more effective than targeting a single biomarker.

According to a previous study, APOBEC-mediated mutagenesis had a significant correlation with APOBEC mRNA levels, particularly A3B [9]. This study revealed that the single nucleotide polymorphism, C → T transitions in cervical, bladder, lung, head and neck, and breast cancers was positively correlated with A3B overexpression, consistent with other previous studies [9, 10, 17]. Moreover, we demonstrated a positive correlation between A3B and MSI. A3B has been shown to induce mutations. Furthermore, microsatellite instability is due to mutations in some mismatch repair genes, such as hMLH1, hPMS2, hMSH2, and hMSH6 [18].

Previous studies have shown that APOBEC enzymes play a role in the innate immune response, mainly in host defense against exogenous viruses and endogenous retrofactors [19, 20]. Surprisingly, APOBEC enzymes have been discovered in immune responses. According to Xia et al., APOBEC3B is a potential biomarker for predicting response to immunotherapy and survival in gastric cancer patients. In particular, APOBEC3B ^high^ CD8+ T cell ^high^ gastric cancer patients were more likely to benefit from adjuvant chemotherapy (ACT) and PD-1 blockade [21]. Hot tumors tend to have a higher immune cell infiltration and immune checkpoint expression [22]. Hot tumors show a reliable response to immunotherapy. Conversely, treatment of cold tumors with immunotherapy remains challenging. According to the present study, the A3B high expression group had a large amount of immune cell infiltration, including CD8+ T cells, B cells, macrophages, and NK cells. Furthermore, immune cells showed a high expression of immune checkpoints such as CD274, HAVCR2, and TIGIT. These results demonstrate a significant association between A3B and immunotherapy.

Several studies have investigated composite biomarkers. According to Yan et al., the composite biomarkers, NKG2A and PD-L1 in muscle-invasive bladder cancer were effective in predicting response to PD-L1 inhibitors and cisplatin-based ACT chemotherapy regimen [23]. Furthermore, the IMvigor210 immunotherapy clinical trial revealed that targeted therapy against the combined biomarkers of A3B and CD274 was more effective than that targeting CD274 alone.

This study did not validate the findings of the IMvigor210 immunotherapy clinical trial. Therefore, further studies should be conducted to validate the findings of this study. In our view, APOBEC3B is a promising target for cancer therapy, just like Zou et al.'s study [24].

## 5. Conclusion

This study demonstrated that the combined biomarker of APOBEC3B and CD274 was more effective in predicting the response to PD-1/PD-L1 inhibitors than a single biomarker of CD274 in bladder urothelial carcinoma. In addition, the study revealed that APOBEC3B was positively correlated with TMB and MSI.

## Data Availability

The original contributions presented in the study are included in the article/supplementary materials. Further inquiries can be directed to the corresponding authors.

## Supplementary Materials

Supplementary 1 Table S1. Immunophenotyping data in pan-cancer.

Supplementary 2 Table S2 Immune checkpoint related genes.
